# Subtle Population Genetic Structure in Yelloweye Rockfish (*Sebastes ruberrimus*) Is Consistent with a Major Oceanographic Division in British Columbia, Canada

**DOI:** 10.1371/journal.pone.0071083

**Published:** 2013-08-21

**Authors:** Matthew R. Siegle, Eric B. Taylor, Kristi M. Miller, Ruth E. Withler, K. Lynne Yamanaka

**Affiliations:** 1 Department of Zoology, Biodiversity Research Centre and Beaty Biodiversity Museum, University of British Columbia, Vancouver, British Columbia, Canada; 2 Pacific Biological Station, Fisheries and Oceans Canada, Nanaimo, British Columbia, Canada; University of Otago, New Zealand

## Abstract

The boundaries between oceanographic domains often function as dispersal barriers for many temperate marine species with a dispersive pelagic larval phase. Yelloweye rockfish (*Sebastes ruberrimus*, YR) are widely distributed across the northeastern Pacific Ocean, inhabiting coastal rocky reefs from the Aleutian Islands in Alaska through southern California. This species exhibits an extended pelagic larval duration and has the capacity for long distance larval transport. We assayed 2,862 YR individuals from 13 general areas in the northeast Pacific Ocean for allelic variation at nine microsatellite loci. Bayesian model-based clustering analyses grouped individuals from the Strait of Georgia (SG) into a distinct genetic cluster, while individuals from outer coastal water locations (OCLs) were partitioned equally across two genetic clusters, including the cluster associated with the SG fish. Pairwise F_ST_ values were consistently an order of magnitude higher for comparisons between the SG and OCLs than they were for all OCL-OCL comparisons (∼0.016 vs. ∼0.001). This same pattern was observed across two time points when individuals were binned into an “old” and “young” group according to birth year (old: ∼0.020 vs. 0.0003; young: ∼0.020 vs. ∼0.004). Additionally, mean allelic richness was markedly lower within the SG compared to the OCLs (8.00 vs. 10.54–11.77). These results indicate that the Strait of Georgia “deep-basin” estuary oceanographic domain acts as a dispersal barrier from the outer coastal waters via the Juan de Fuca Strait. Alternatively, selection against maladapted dispersers across this oceanographic transition may underlie the observed genetic differentiation between the Georgia basin and the outer coastal waters, and further work is needed to confirm the SG-OCL divide acts as a barrier to larval dispersal.

## Introduction

Population replenishment for a large number of marine populations depends upon the input of exogenously derived individuals [1]–[4], which can occur via an extended pelagic larval phase [5], [6]. The cumulative effects of larval trajectory, planktonic survival, delivery to settlement habitat and postsettlement performance result in variable contributions in local larval input from outside sources [7], [8]. Mismatches between the spatial scales of exploited management units and of population replenishment may precipitate overexploitation of targeted species [9]–[11]. Thus, effective management of harvested species requires an understanding of the pattern of larval dispersal [12]. Furthermore, the utility of marine protected area (MPA) networks increases with their ability to enhance net larval production and export into exploitable areas while remaining self-sufficient via larval input from upstream MPAs [13]–[15].

Oceanography is an important driver of population structure in temperate reef fishes, as ocean currents largely dictate larval trajectories, planktonic survival and delivery to settlement habitat [7], [16]. Offshore advection of larvae or propagules may lead to long distance larval transport [17], [18]. On the other hand, there are a number of retention mechanisms that limit dispersal distances [18]–[22]. These include fronts associated with upwelling [23] or the intersection of independent current systems [24], gyres that form around seamounts or other complex bathymetric features [25]–[27], and eddies [28], [29], including those formed by currents moving around rugged coastlines [30], [31]. In lieu of directly measuring dispersal distances, which is often difficult or intractable [32], population genetic structure can inform indirect estimates of the scale of dispersal [33]–[36]. Genetic structure that occurs across oceanographic features suggests that these features may function as long-standing barriers to dispersal [37]–[39], influencing the spatial scale of ecological and evolutionary processes.

Rockfishes (*Sebastes* spp.) constitute a diverse group of nearshore fishes that are distributed in temperate waters around the world [40]. The bulk of the diversity (∼65 species) occurs in the northeast Pacific Ocean, where rockfishes are found in every habitat type from the intertidal waters to depths greater than 1,500 meters [40]. This group shares many life-history attributes, such as slow maturation rate and highly variable juvenile recruitment that contribute to their low productivity and make them susceptible to overfishing [41]. Rockfishes are characterized by large populations, high fecundities, and pelagically dispersed larvae with the potential for widespread gene flow. Population genetic studies of rockfishes have shown that despite an extended pelagic larval duration (PLD), they often exhibit population structure over regional scales, potentially explained by the absence of settlement habitat [42] or concordant oceanographic divisions [43]–[49]. For example, Point Conception in southern California is associated with a genetic break in blue rockfish (*S. mystinus*) [47] and vermillion rockfish (*S. miniatus*) [50], while both copper rockfish (*S. caurinus*) and brown rockfish (*S. auriculatus*) from the Puget Sound basin exhibit significant genetic divergence from populations located along the outer coast [45], [48], [51].

In this study, we used microsatellite genetic markers to assess the population genetic structure of yelloweye rockfish (*S. ruberrimus;* YR) in the northeast Pacific Ocean. We predicted that population structure would occur across oceanographic features that likely function as barriers to dispersal, resulting in three genetically differentiated subdivisions. These predicted genetic subdivisions include (i) the separation of the Georgia basin from the outer coast via the Juan de Fuca Strait and (ii) isolation of the Bowie Seamount location. We also predicted an isolation by distance signal would be detected across the outer coast locations, which span over 1,500 km of coastline. We detected subtle genetic structure, partitioning the Strait of Georgia population from a panmictic outer coast population. No signal of isolation by distance was detected across the outer coast sampling locations.

## Materials and Methods

### Study area and study species

Water circulation patterns in the northeast Pacific Ocean are largely structured by the eastward flowing Subarctic Current, which divides into the southern flowing California Current and northern flowing Alaska Current well offshore of Vancouver Island, British Columbia [52]. In British Columbia, the nearshore waters of the outer coast are dominated by the directional flow of the Davidson and Vancouver Island coastal currents, which dominate in winter and summer, respectively [53]. Circulation patterns within the inshore waters of the Georgia basin are typified by tidal currents and, in the summer, estuarine currents caused by freshwater input from the Fraser River [52], [54]. Tidal forces cause intense vertical mixing of brackish surface waters and deep water from the Juan de Fuca Strait along sills located throughout the San Juan Archipelago and southern Georgia basin [54]. Through the vertical forcing of deep water and subsequent mixing that occurs with surface waters, these sills may act as physical barriers to larval dispersal [55], [56].

Yelloweye rockfish are one of the larger and longest-lived species of rockfishes, reaching lengths greater than 80 cm and often living longer than 100 years [40]. Slow maturation rate, highly variable juvenile recruitment success, and long lifespans contribute to YR's susceptibility to overfishing [41], [57], and conservation concerns in Canada and the United States have precipitated formal status assessments by The Committee on the Status of Endangered Wildlife in Canada (COSEWIC) and the National Marine Fisheries Service. This species is currently listed as “Special Concern” and “Threatened” in British Columbia and Puget Sound, respectively [58], [59].

### Sample collection

**Figure 1:**
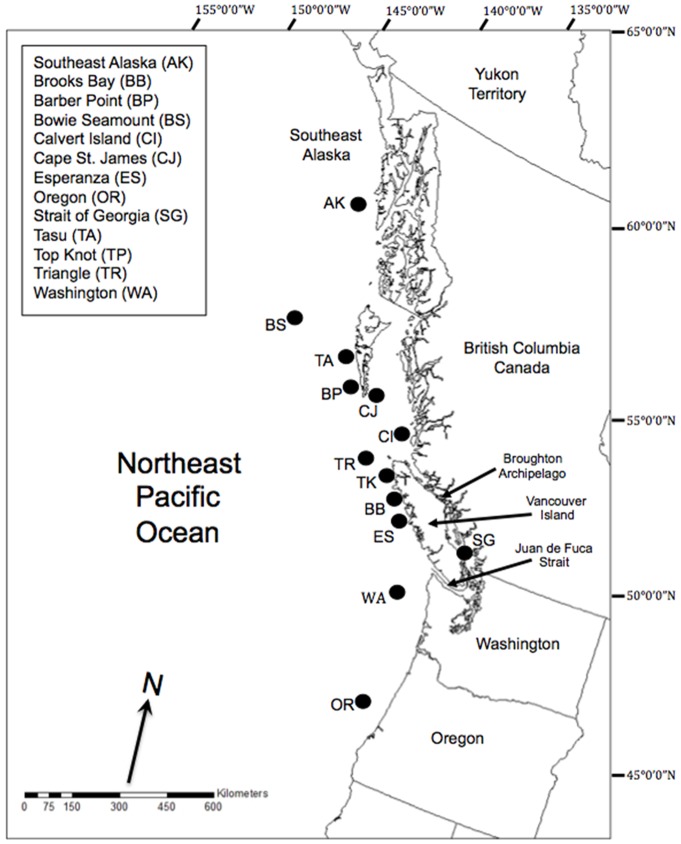
Map of sampling locations in Northeast Pacific Ocean. Sampling locations and location codes of yelloweye rockfish (*Sebastes ruberrimus*), assayed at nine microsatellite loci.

Yelloweye rockfish tissue samples were collected from 13 general areas in the coastal waters of British Columbia (including Bowie Seamount, approximately 200 km west of Haida Gwaii), southeast Alaska, Washington and Oregon (Figure 1, Table 1). Fish were sampled during Fisheries and Oceans Canada inshore rockfish stock assessment longline surveys, and opportunistically from commercial fishery vessels from 1998–2006. The majority of sample locations are comprised of individuals that were collected during a single survey. Strait of Georgia individuals, however, were collected over two years of sampling. Surveys were not restricted to a single season, and samples were collected over all seasons. Although we defined each sample area as a single location, they comprised samples collected from a range of individual sites within the general area of each of the 13 sample areas. Scientific collection permits and animal care approval were not required as tissue samples were taken from individuals already sampled as part of the DFO Inshore Rockfish Research Program, and opportunistically from commercial fishery vessels. Tissue samples were taken either onboard the fishing vessel or dockside and stored in 95% ethanol for genetic analyses. Sagittal otoliths were taken from a subset of individuals and locations for ageing.

**Table 1:** Sample location specific descriptive statistics.

Sample Location	Code	N	Mean A_R_	H_O_	H_E_	F_IS_	P-value
Southeast Alaska	AK	85	11.13	0.711	0.722	0.015	0.2038
Brooks Bay	BB	50	10.59	0.723	0.731	0.012	0.2812
Barber Point	BP	345	11.14	0.711	0.728	0.024	0.0034
Bowie Seamount	BS	779	11.10	0.701	0.726	0.034	**0.0004**
Calvert Island	CI	87	11.15	0.701	0.725	0.033	0.0308
Cape St. James	CJ	327	10.99	0.692	0.723	0.043	**0.0004**
Esperanza	ES	46	10.99	0.725	0.730	0.007	0.3735
Oregon	OR	75	10.54	0.684	0.714	0.043	0.0150
Strait of Georgia	SG	123	8.00	0.630	0.631	0.003	0.4406
Tasu	TA	231	11.32	0.743	0.741	−0.002	0.5795
Top Knot	TK	167	11.21	0.716	0.728	0.017	0.0718
Triangle	TR	187	11.77	0.689	0.728	0.054	**0.0004**
Washington	WA	81	11.12	0.707	0.723	0.024	0.0868

Sample size (N), mean allelic richness (A_R_), observed heterozygosity (H_O_), expected heterozygosity (H_E_), coefficient of inbreeding (F_IS_) and associated P-values are shown for each sample location. P-values in **bold** denote significant heterozygote deficiencies at the 5% level after correcting for multiple comparisons (critical value: 0.00043).

### Laboratory methods and scoring

Total genomic DNA was extracted with Qiagen Dneasy extraction kits (Qiagen, Valencia, California). Nine microsatellite loci (Table S1 in File S1) were amplified using polymerase chain reaction (PCR). Typical PCR cycling conditions included an initial denaturation at 94°C for 2 min, followed by ∼30 cycles of 94° for 1 min, 46°–60° for 30 sec, a 72° extension for 1 min, and a final extension at 72° for 10 min. Annealing times and temperatures were adjusted to optimize specific locus amplification. Forward PCR primers were fluorescently labeled and fragment sizing was conducted on the ABI 377 automated DNA sequencer platform (Applied Biosystems (ABI), Foster City, California). Fragments were sized using the GeneScan-500 size standard and allele scoring was performed with GENEMAPPER version 3.7 (ABI).

### Data analysis

Deviations from Hardy-Weinberg equilibrium were assessed for each locus-population combination with GENEPOP version 3.1 [60]. Estimates of the exact P-values were obtained using the Markov chain method (1000 batches, 5000 iterations per batch). Genotypic linkage disequilibrium for all combinations of locus pairs within sample locations was calculated in GENEPOP (100 batches, 1000 iterations per batch). Measures of heterozygosity and allelic richness were calculated in FSTAT version 2.9.3.2 [61]. The assumption of selective neutrality was assessed with the selection detection workbench LOSITAN [62], which implements the F_ST_ outlier approach of FDIST [63]. Tests for isolation-by-distance (IBD) were performed with Mantel tests using the *ade4* package [64] in the R environment [65]. Pairwise F_ST_ values were linearized (F_ST_/1-F_ST_) according to Rousset [66].

Population structure was assessed with estimates of the summary statistic, F_ST_, as well as with the genetic clustering program STRUCTURE version 2.3 [67]–[69]. Pairwise F_ST_ values were estimated with θ [70] using the permutation approach implemented in FSTAT. To test for temporal stability of pairwise F_ST_ values, 420 individuals (which have accompanying age data) from eight sample locations were binned into a “young” or “old” age group according to birth year. Individuals born before and after 1980 were binned into the “old” and “young” group, respectively. The year 1980 was chosen as the cutoff as it is the median birth year, which also maximizes sample size similarity between the sample locations for each age group (Table S2 in File S1). STRUCTURE was used to determine the number of distinct genetic clusters (*K*) among the sample locations, and to estimate individual assignment probabilities for each fish to each resolved cluster. The number of putative genetic clusters assessed ranged from 1 to 7. Each run consisted of a 500,000 step burn-in plus an additional 1,000,000 steps, and 20 iterations were run for each *K*-value. Due to low overall genetic structuring (global F_ST_  = 0.002), the number of genetic clusters was evaluated under both an admixture model including a location prior [69] in addition to an admixture model without a location prior. We did not utilize the method of Evanno et al. [71], which calculates the second order rate of change (Δ*K*) as an estimator of *K*, due to the limited success of this method observed when overall differentiation is low [72].

## Results

### Genotyping and scoring

A total of 2862 individuals from 13 general sample locations (sample sizes ranged from 46 to 779) were assayed at nine microsatellite loci. Only individuals with allele scores from a minimum of seven loci were retained for analysis. The majority of individuals (66.8%) had genotype scores across all nine loci, 628 (21.9%) contained missing data for one locus, and 323 (11.3%) individuals had missing data for two loci. Missing data, therefore, constitutes only 4.9% of the total dataset. There was no evidence for selection acting at any of the microsatellite loci using LOSITAN (for all loci P>0.24; data not shown).

### Within population variation

The number of alleles across all populations ranged from 10 (*Sal3*) to 39 (*Sme3*) with an average of 18.9 alleles per locus. Expected heterozygosity ranged from 0.488 (*Sru9*) to 0.877 (*Sal1*) and averaged 0.702 across loci and sample locations (Table S3 in File S1). A relatively high proportion of samples (24 out of 117 comparisons) were found to be out of Hardy-Weinberg equilibrium (HWE) with a critical value of 0.05. After using the Bonferroni correction for multiple comparisons, however, the number of departures from HWE dropped to eight (critical value: 0.0004). Samples exhibiting a departure from HWE were distributed across sample locations and loci, and, therefore are unlikely to affect our results because they do not point to consistently anomalous loci or localities. Only 39 (out of 468) locus-locus within sample location comparisons exhibited significant deviations from linkage equilibrium (P<0.05). These 39 departures were not concentrated on a particular locus pair or within specific sample locations, and are also unlikely to affect our results.

### Among population variation

The amount of genetic variation attributable to differences between sample locations was low (global F_ST_  = 0.002, *P*<0.01). Pairwise F_ST_ values range from less than 0 to 0.0193, and were consistently an order of magnitude higher for all comparisons between the Strait of Georgia (SG) sample location and outer coastal water locations (OCLs) than for comparisons between OCLs (∼0.016 vs. ∼0.001; Table 2). This same pattern was observed when comparisons were restricted to both the “old” and “young” groups (mean value for old SG-OCL vs. old OCL-OCL: ∼0.0203 vs. ∼0.0003; mean value for young SG-OCL vs. young OCL-OCL: 0.0204 vs. 0.0041; Table S4 and Table S5 in File S1). Furthermore, mean (across loci and locations) allelic richness was markedly lower in the SG location compared to the OCLs (8.00 vs. 10.54–11.77, average for OCLs: 11.10; Table 1).

**Table 2:** Pairwise F_ST_ values.

	AK	BB	BP	BS	CI	CJ	ES	OR	SG	TA	TK	TR	WA
AK	–												
BB	0.0002	–											
BP	−0.0001	0.0019	–										
BS	0.0004	0.0003	0.0005	–									
CI	0.0033	0.0012	0.0015	0.0012	–								
CJ	0.0005	0	0.0002	−0.0001	0.0007	–							
ES	0.0006	0.0002	−0.0002	−0.0011	0.0010	−0.0018	–						
OR	0.0036	0.0012	0.0016	0.0006	−0.0004	0.0012	0.0013	–					
SG	**0.0163**	**0.0189**	**0.0156**	**0.0165**	**0.0175**	**0.0142**	**0.018**	**0.0193**	–				
TA	0.0003	0.0002	**0.0011**	0.0002	0.0018	0.0009	−0.0001	0.0018	**0.0193**	–			
TK	0.0009	0.0006	0.0005	0.0004	0.0006	0.0003	0.0014	0.0007	**0.0138**	0.0001	–		
TR	0.0015	0.0004	0.0008	0.0003	0.0013	0.0006	0.0007	0.0016	**0.0168**	0.0011	0.0001	–	
WA	−0.0005	−0.0023	0.0010	−0.0001	0.0010	−0.0003	0.0002	0.0018	**0.0164**	0.0009	0.0008	0	–

Pairwise F_ST_ values for all sample locations are shown. Values in **bold** type are significant after correcting for multiple comparisons using the Bonferroni correction (adjusted critical value: 0.000641). All pairwise comparisons with the Strait of Georgia (SG) sample location are significant, and amongst the outer coast location comparisons, only the Barber Point (BP) – Tasu (TA) pairwise comparison is significant.

**Figure 2:**
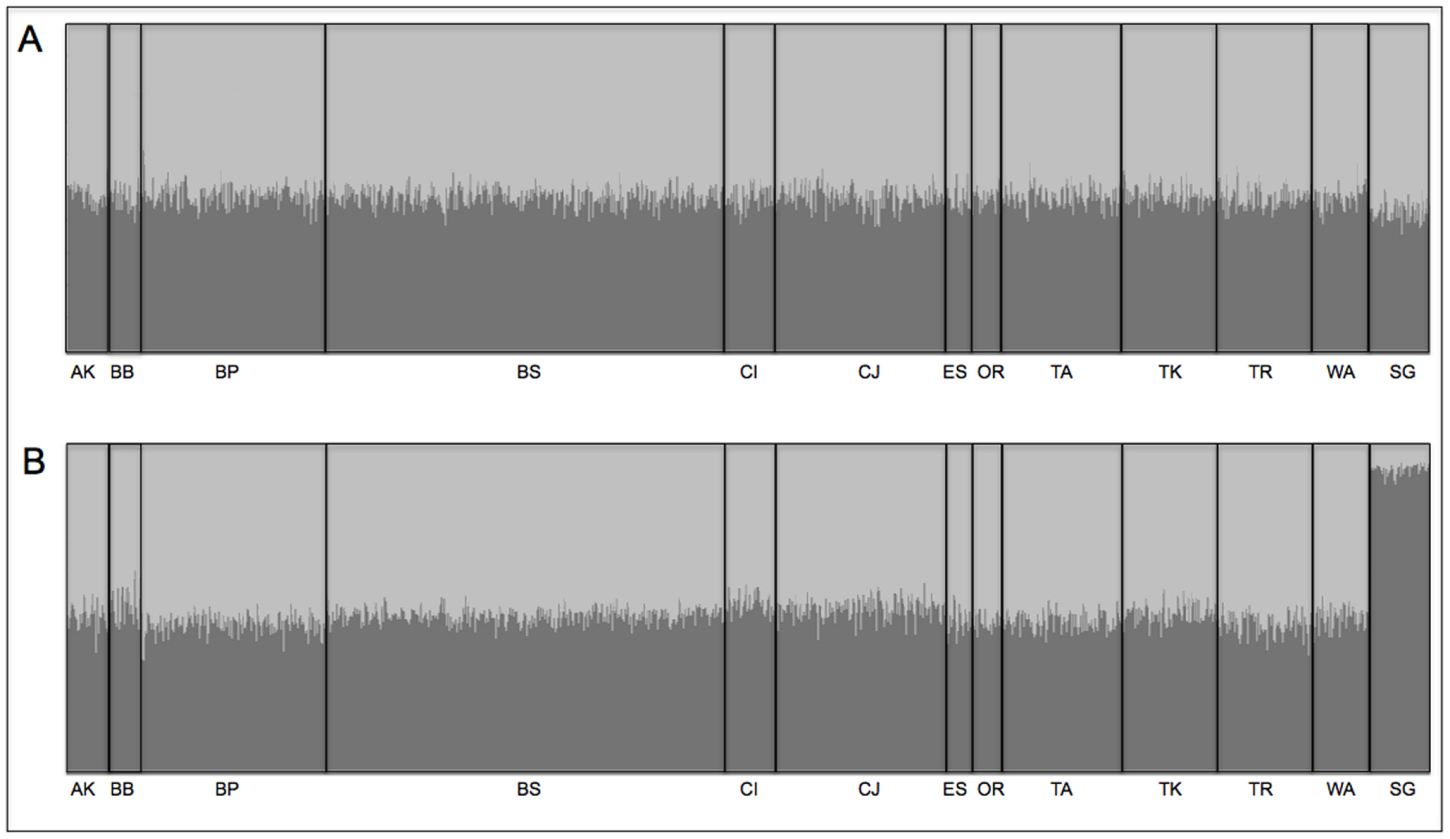
STRUCTURE analysis. STRUCTURE outputs for the admixture model without a location prior (*a*) and the admixture model with the location prior (*b*). The genome of each individual fish is represented by a thin vertical line as assayed by nine microsatellite markers, where each shade of grey represents a unique genetic cluster, and the proportion of each genetic cluster that contributes to an individual's genome is illustrated by the relative amount of each shade within each vertical line. Under the admixture model without a location prior, all individuals exhibit roughly equal admixture between the two genetic clusters (q is approximately 0.5). Under the admixture model with a location prior, however, the Strait of Georgia individuals exhibit a q-value close to 1.0, while the outer coast location individuals still exhibit equal admixture of both clusters.

Two admixture models, one without a location prior and one with a location prior were evaluated using STRUCTURE. The results based on the model without the location prior failed to detect more than a single genetic cluster (highest support for a *K* of 1) among our samples. By contrast, results from the model including a location prior had equal support for a *K* of 1 and a *K* of 2 (Table S6 in File S1), yet SG individuals exhibited high q-values (minimum is 0.87, mean is 0.93), while all OCL individuals exhibited approximately equal admixture of both genetic clusters (q-value approximately equal to 0.5; Figure 2).

**Figure 3:**
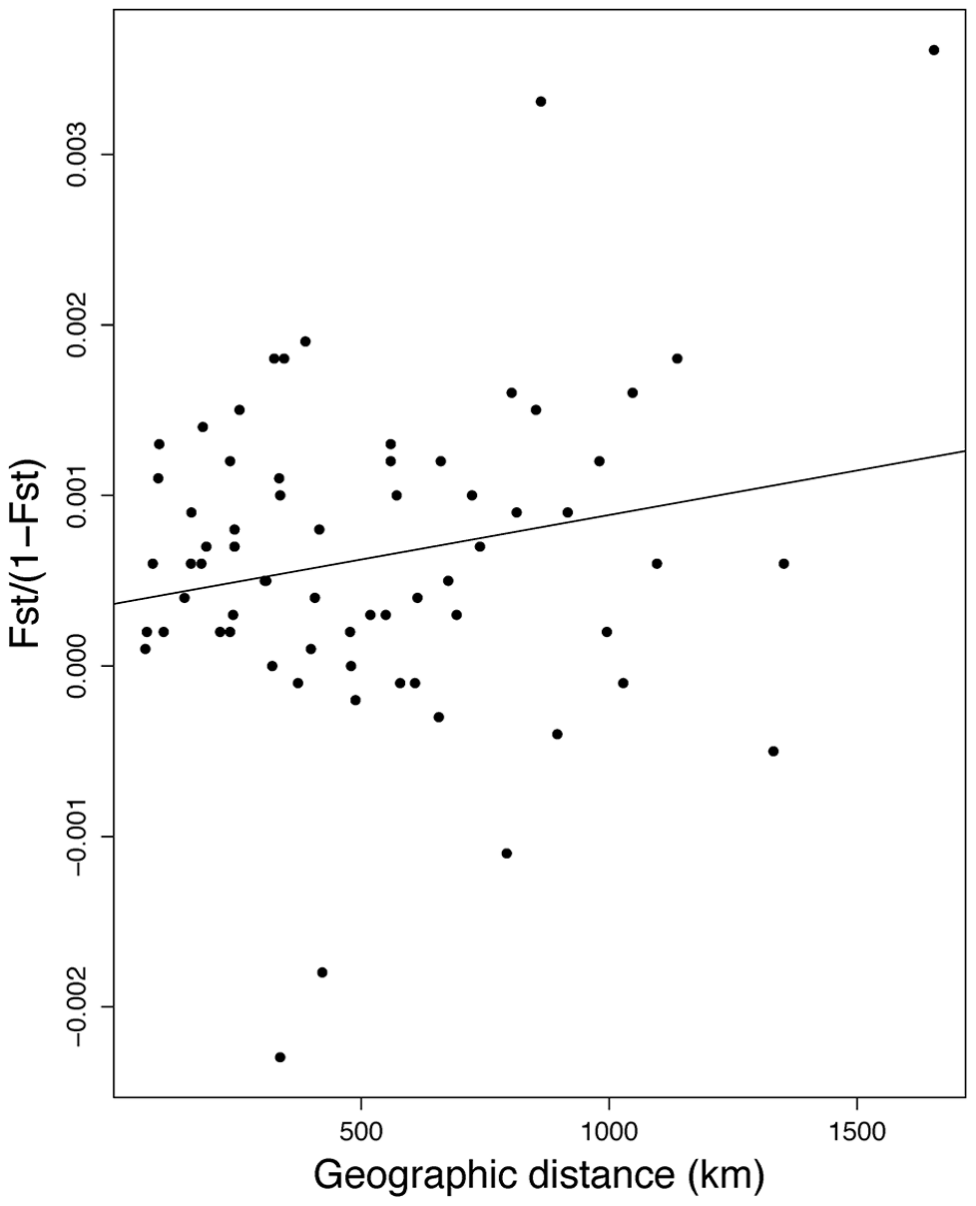
Isolation-by-distance analysis. The relationship between genetic distance (F_ST_/1-F_ST_) and geographic distance is shown for all pairwise locations, excluding the Strait of Georgia and Bowie Seamount sample locations. No significant correlation was detected with a Mantel test (P>0.25).

No significant association between genetic distance and geographic distance was detected with the Mantel tests (Figure 3). Two IBD analyses were conducted, one including all pairwise sample location comparisons (Mantel *P* = 0.250) and one with the Strait of Georgia and Bowie Seamount sample locations removed (Mantel *P* = 0.268).

## Discussion

### Population genetic structure in yelloweye rockfish

We detected subtle population genetic structure that separates a putative Strait of Georgia population from a panmictic outer coast population. The results presented here constitute a re-analysis of YR population genetic structure originally investigated by Yamanaka et al. [73] and Yamanaka et al. [74]. Yamanaka et al. [73] present genetic data from several OCLs (including fish from Bowie Seamount), and failed to reject the hypothesis that all individuals were derived from a single, panmictic population. Yamanaka et al. [74] extended the sampling to include fish from the SG, and, using tree-based analyses, identified a putative SG-OCL genetic break with moderate bootstrap support. Our analyses support this putative population boundary, and provide some support for the temporal stability of this population structure. While increasing the number of sample locations within the Strait of Georgia, as well as the number of temporal comparisons would bolster the robustness of the population structure we observed, similar results were found in a separate analysis conducted with a different molecular marker type, Amplified Fragment Length Polymorphisms [75] and with results for other rockfishes [45], [48], [51].

### Natural selection as a mechanism promoting genetic isolation of the Georgia basin

One interpretation of the genetic structure observed between the SG and the OCLs is that the oceanographic transition between these areas functions as a physical barrier to larval dispersal. Alternatively, dispersal may occur across this oceanographic divide and natural selection against maladapted dispersers reduces connectivity and promotes genetic differentiation [8]. The Georgia basin is a unique environment, and likely presents individuals with varying selective forces [52]. Selection, however, is more likely to differentiate a subset of functionally important loci [45], while the genetic differentiation we observed was due to the combined effects of all (putatively neutral) loci, rather than being driven by a single locus with a large effect. This suggests that a genome-wide process, such as reduced gene flow, underlies the observed genetic structure. If selection were driving this pattern, we would expect it to be operating on many loci spread across the genome in order to affect all nine of the microsatellite markers via hitchhiking, a scenario less consistent with our data.

### Lack of genetic structure in the outer coastal waters

Bowie Seamount (58.23°N 135.74°W) is approximately 200 kilometers west of Haida Gwaii and rises to within 30 meters of the surface from depths over 3,000 meters creating rockfish habitat that is isolated from coastal areas [52]. Despite these putative barriers to larval exchange, we found no evidence for genetic differentiation of the Bowie Seamount sample from other outer coast sample locations. Episodic recruitment of larvae from coastal populations to Bowie Seamount may be driven by Haida eddies [76]–[79], mesoscale vortices that form along the west coast of Haida Gwaii and move westward into the Gulf of Alaska and may persist for several years [76], [77]. While the PLD of YR is unknown, the PLDs of other rockfishes ranges from one to several months, but may be up to one year [40]. Given the average velocity of a Haida eddy, pelagic larval durations on the longer side may allow for sufficient transport time to Bowie Seamount, which may help explain the apparent genetic homogeneity we observed in this study.

The apparent lack of genetic structure or a significant IBD relationship between the OCLs should not be taken, on its own, as evidence supporting high connectivity between these locations. High effective population sizes (Ne), commonly observed in marine fishes, may resist the effects of drift and contribute to low F_ST_ values [80]. Using estimates of population genetic structure to infer contemporary patterns of gene flow (and migration rates) may be problematic, as these estimates reflect both historical and contemporary patterns of gene flow, [81]. Over evolutionary time, one migrant per generation can be sufficient to homogenize neutral allele frequencies [82], [83]. Thus, rare historical dispersal events may be sufficient to confound contemporary patterns of gene flow, but not reflect ecologically relevant influx of larvae. Moreover, consistent dispersal following a stepping-stone pattern over long time scales may lead to genetic homogenization.

### Management and conservation of yelloweye rockfish

Conservation concerns surrounding Pacific rockfishes (*Sebastes* spp.) in the late 1990's [41] precipitated a number of changes to the management of inshore rockfish in British Columbia. In 2002, Fisheries and Oceans Canada (DFO) introduced a Rockfish Conservation Strategy to address these concerns and slow apparent population declines. Four specific measures were implemented: (i) account for all rockfish catch, (ii) decrease fishing mortality, (iii) establish areas closed to fishing, and (iv) improve stock assessment and monitoring (for details please see [53]). Concurrent with the implementation of the Rockfish Conservation Strategy, YR were reviewed by COSEWIC [58] to determine if available information warranted a protected status. Based on the genetic and demographic differences identified by Yamanaka et al. [74] and supported by this study, two Designatable Units (DUs) of YR were delineated and assessed by COSEWIC. Both the inside and outside DUs (SG and outer coastal waters, respectively) are listed as “Special Concern”, owing mainly to the life-history characteristics (e.g., long-life spans, slow maturation rate, and highly variable juvenile recruitment) that make YR susceptible to overfishing and have the potential to slip into “Threatened” status [58].

As part of the Rockfish Conservation Strategy, areas suitable for protection were identified through a series of consultations and habitat modelling [57]. The first rockfish conservation areas (RCAs) were designated in 2002, and at present there are 164 RCAs in the coastal waters of British Columbia (Figure S1 in File S1). The RCAs constitute approximately 20% and 30% of outer coast and Georgia basin rockfish habitat, respectively. While the implementation of the RCAs constitutes a significant achievement in marine conservation, much work remains to understand how these protected areas affect demographic and ecological processes within the greater region.

Understanding larval movement is a critical component in understanding the regional effect of marine protected areas (MPAs) [84], [85]. Larvae originating within protected areas may either recruit locally, enhancing populations within the MPA or spill over MPA boundaries, enhancing outside fished areas. The extent to which MPAs are self-sufficient (i.e. self-recruiting) depends upon the size of the MPA in relation to the dispersal distances of locally produced larvae. This is an important consideration for the RCAs, as they were not delineated to purposefully function as a network connected by larval dispersal. Furthermore, there is considerable variation in the size and spacing of RCAs between those located in inside and outside waters. Future studies will need to assess the number of individuals residing in outside and inside RCAs to understand how larval output from RCAs may vary between outside and inside areas, and what effect that entails for the role of upstream RCAs acting as larval sources for downstream RCAs.

## Conclusion

Our analyses support a population boundary between the inshore waters of the Strait of Georgia and the outer coastal waters that coincides with the transition between two oceanographic domains via the Juan de Fuca Strait. Several important implications can be drawn from this result. Principally, the genetic data suggest that dispersal is restricted regionally by major oceanographic features. This scale of dispersal seems to match the scale at which YR stocks are managed, although more detailed studies are needed to fully elucidate the complex metapopulation dynamics observed. If dispersal between the Strait of Georgia and the outer coastal waters is indeed rare or demographically insignificant, it is unlikely that outside areas will function as substantial larval sources for the inshore population.

## Supporting Information

File S1 **Supporting figures and tables.** Figure S1. Rockfish Conservation Areas. The distribution of rockfish conservation areas (RCAs) in British Columbia (figure reproduced with permission from Yamanaka & Logan [57]). Information about the RCAs can be found on the DFO website: http://www.pac.dfo-mpo.gc.ca/fm-gp/maps-cartes/rca-acs/index-eng.htm. Table S1. Primer information. The forward (F) and reverse (R) primer sequences, PCR annealing temperatures (T_A_), Genbank accession number (GB AC #), and reference for each microsatellite locus are shown below. Table S2. Temporal population structure comparisons. The sample size for the “old” and “young” datasets (N_old_, N_young_), as well as the pairwise F_ST_ value for each within location, “old” vs. “young” comparison is shown below. None of the pairwise F_ST_ values is statistically significant. Table S3. Individual locus descriptive statistics. Mean number of alleles (N_A_), total number of alleles (N_T_), observed heterozygosity (H_O_), expected heterozygosity (H_E_), inbreeding coefficient (F_IS_), theta, and standard error of theta (S.E.) for each locus are shown. Table S4. “Old” pairwise F_ST_ values. Pairwise F_ST_ values for the “old” dataset are shown below. Statistically significant values are shown in **bold**. Table S5. “Young” pairwise F_ST_ values. Pairwise F_ST_ values for the “young” dataset are shown below. Statistically significant values are shown in **bold**. Table S6. STRUCTURE mean log likelihood results. Mean log likelihood of each *K* value (LnP(*K*)) and standard deviation (S.D.) are shown for both models evaluated in STRUCTURE: an admixture model without a location prior, and an admixture model with a location prior. The most likely value of *K* is shown in **bold** under each model.
(DOC)
