# Supplementary material for: Subtle Population Genetic Structure in Yelloweye Rockfish (Sebastes ruberrimus) Is Consistent with a Major Oceanographic Division in British Columbia, Canada
Source: PLoS One. 2013 Aug 21;8(8):e71083. doi: 10.1371/journal.pone.0071083 (PMC3749191; doi:10.1371/journal.pone.0071083)
Supplement: File S1 — Supporting figures and tables. Figure S1. Rockfish Conservation Areas. The distribution of rockfish conservation areas (RCAs) in British Columbia (figure reproduced with permission from Yamanaka & Logan [57]). Information about the RCAs can be found on the DFO website: http://www.pac.dfo-mpo.gc.ca/fm-gp/maps-cartes/rca-acs/index-eng.htm. Table S1. Primer information. The forward (F) and reverse (R) primer sequences, PCR annealing temperatures (TA), Genbank accession number (GB AC #), and reference for each microsatellite locus are shown below. Table S2. Temporal population structure comparisons. The sample size for the “old” and “young” datasets (Nold, Nyoung), as well as the pairwise FST value for each within location, “old” vs. “young” comparison is shown below. None of the pairwise FST values is statistically significant. Table S3. Individual locus descriptive statistics. Mean number of alleles (NA), total number of alleles (NT), observed heterozygosity (HO), expected heterozygosity (HE), inbreeding coefficient (FIS), theta, and standard error of theta (S.E.) for each locus are shown. Table S4. “Old” pairwise FST values. Pairwise FST values for the “old” dataset are shown below. Statistically significant values are shown in bold. Table S5. “Young” pairwise FST values. Pairwise FST values for the “young” dataset are shown below. Statistically significant values are shown in bold. Table S6. STRUCTURE mean log likelihood results. Mean log likelihood of each K value (LnP(K)) and standard deviation (S.D.) are shown for both models evaluated in STRUCTURE: an admixture model without a location prior, and an admixture model with a location prior. The most likely value of K is shown in bold under each model. (DOC) [file pone.0071083.s001.doc]

**Supporting Information**

Figure S1

**
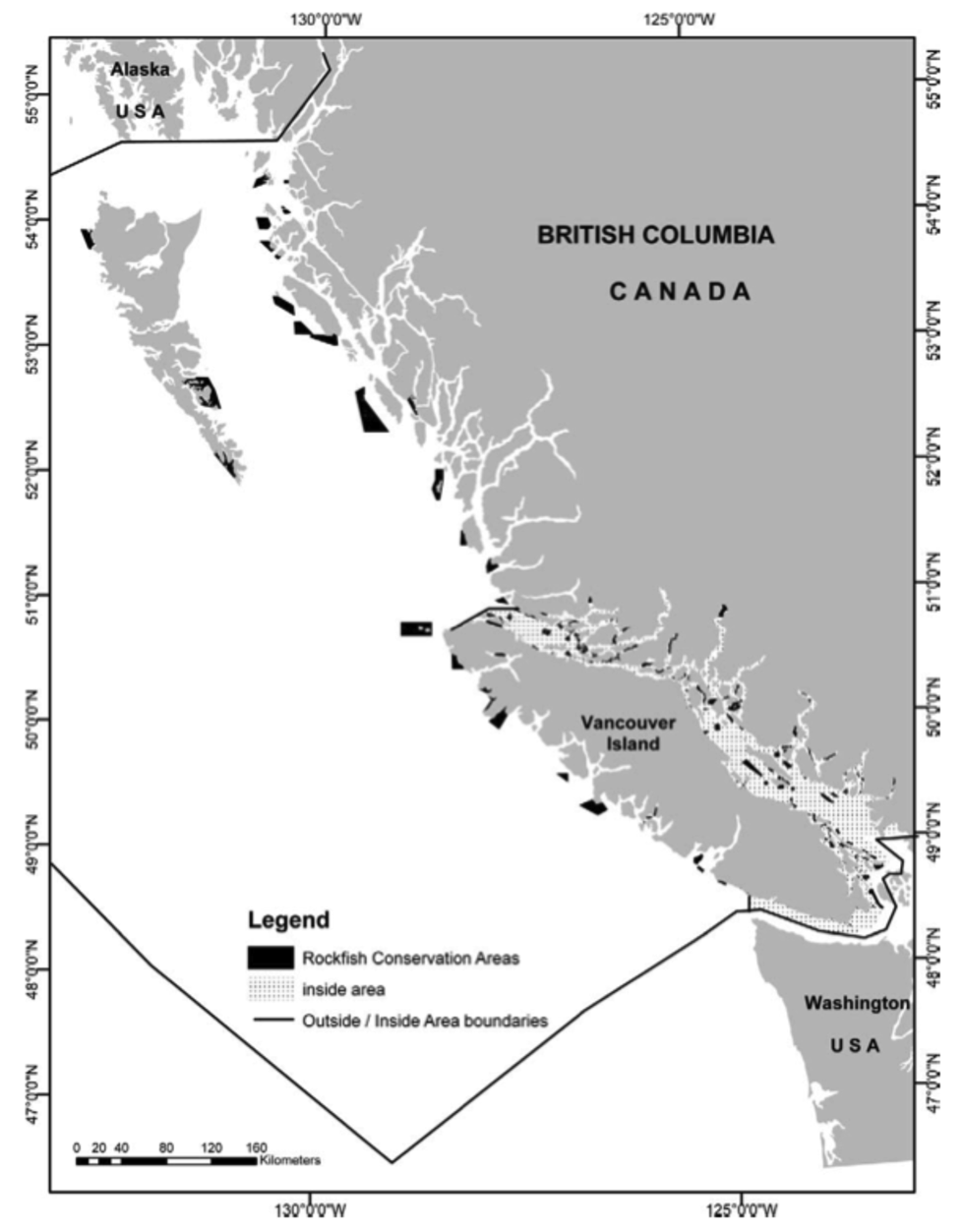
**

Table S1

| Locus | T_A_ (°C) | F primer (5’ – 3’) | R primer (5’ – 3’) | GB AC # | Reference |
| --- | --- | --- | --- | --- | --- |
| *Sal1* | 45 | GTTTGATTTCTACCTTTAGA | GTTTCAGTAATTAAAAGA GAATAAGG | AF153595 | 1 |
| *Sal3* | 46 | GTAGCTTCTTTACAATGAA | GTTTACAGCAGACAGCAGTTCT | AF153597 | 1 |
| *Sme3* | 59 | GTTTGCTTTCTGGAGAAGTCGAGT | GGCATCACTTTCTCTACATAAC | AF142485 | 2 |
| *Sme5* | 56 | GTTTCCAAATTCATCTCAGAACTCT | CCCTTGGGATCAATAAAGTTA | AF142487 | 2 |
| *Sme8* | 53 | GGCTAGTGTGGTATCACTATA | TCCCTCGGGATCAATAAAGTT | AF142490 | 2 |
| *Sme12* | 53 | ATGGGAGAACCGTGATCATA | GTTTGAATTTCCCTCAGGATCAA | AF142494 | 2 |
| *Sme13* | 60 | CTCGGGATCGATAAAGTTAC | GTTTCTGGTTCATACTTGTATTTTGTGTT | AF142495 | 2 |
| *Sru9* | 50 | GAAGATTTCAGGTGACTCTGAAT | GTTTGTACCTCACCATCTTCCA | AEF032500 | 3 |
| *Sru20* | 46 | TGTACTGTAGCTCTCTCATT | GTTTCCAAAGCTAAGATACTTGTTTGTAA | --- | 4 |

^1^Miller, K.M., A.D. Schulze, and R.E. Wither (2000) Characterization of microsatellite loci in *Sebastes alutus* and their conservation in congeneric rockfish species. *Molecular Ecology* 9(2), 240-242.

^2^Seeb, L.W., E.J. Kretschmer, and J.B. Olsen (1999) Characterization of microsatellite loci derived from Black Rockfish (*Sebastes melanops*)*. unpublished*

^3^Miller, K.M., A.D. Schulze, and R.E. Withler (2006) A novel approach to individual and species identification of rockfish. *unpublished*

^4^Miller, K.M, A.D. Schulze, and R.E. Withler (2000) *unpublished*

Table S2

| Location | N_old_ | N_young_ | Within location, old vs. young pairwise F_ST_ |
| --- | --- | --- | --- |
| AK | 40 | 10 | -0.0041 |
| BS | 7 | 43 | 0.0012 |
| CI | 19 | 31 | -0.0044 |
| CJ | 14 | 36 | 0.0017 |
| ES | 19 | 28 | 0.001 |
| SG | 47 | 76 | -0.0027 |
| TK | 22 | 28 | -0.0036 |
| TR | 24 | 26 | 0.0033 |

Table S3

| Locus | N_A_ | N_T_ | H_O_ | H_E_ | F_IS_ | theta | S.E. |
| --- | --- | --- | --- | --- | --- | --- | --- |
| *Sal1* | 24 | 28 | 0.877 | 0.899 | 0.024 | 0.002 | 0.002 |
| *Sal3* | 7.0 | 10 | 0.601 | 0.582 | -0.011 | 0.000 | 0.001 |
| *Sme3* | 23.1 | 39 | 0.560 | 0.585 | 0.016 | 0.004 | 0.004 |
| *Sme5* | 7.9 | 12 | 0.667 | 0.673 | 0.005 | 0.002 | 0.002 |
| *Sme8* | 12.6 | 17 | 0.757 | 0.784 | 0.036 | 0.001 | 0.001 |
| *Sme12* | 14.1 | 16 | 0.803 | 0.863 | 0.069 | 0.001 | 0.002 |
| *Sme13* | 13.3 | 16 | 0.708 | 0.752 | 0.084 | 0.002 | 0.002 |
| *Sru9* | 9.9 | 14 | 0.488 | 0.483 | -0.013 | 0.004 | 0.004 |
| *Sru20* | 13.4 | 18 | 0.860 | 0.855 | 0.006 | 0.000 | 0.001 |
| overall | 13.9 | --- | 0.702 | 0.719 | 0.028 | 0.002 | --- |

Table S4

|  | AK | BS | CI | CJ | ES | SG | TK | TR |
| --- | --- | --- | --- | --- | --- | --- | --- | --- |
| AK | --- |  |  |  |  |  |  |  |
| BS | **0.0187** | --- |  |  |  |  |  |  |
| CI | 0.0015 | -0.0092 | --- |  |  |  |  |  |
| CJ | -0.0042 | 0.0052 | -0.0051 | --- |  |  |  |  |
| ES | -0.0048 | **0.0101** | 0.0016 | 0.0022 | --- |  |  |  |
| SG | **0.019** | **0.0322** | **0.0137** | **0.0289** | **0.0201** | --- |  |  |
| TK | -0.0014 | 0.0019 | -0.0062 | -0.0075 | 0.0052 | **0.0123** | --- |  |
| TR | -0.0006 | 0.0008 | -0.0004 | 0.0009 | -0.0004 | **0.0157** | -0.0021 | --- |

Table S5

|  | AK | BS | CI | CJ | ES | SG | TK | TR |
| --- | --- | --- | --- | --- | --- | --- | --- | --- |
| AK | --- |  |  |  |  |  |  |  |
| BS | 0.0093 | --- |  |  |  |  |  |  |
| CI | **0.0165** | -0.0029 | --- |  |  |  |  |  |
| CJ | 0.0108 | 0.0009 | -0.0009 | --- |  |  |  |  |
| ES | 0.0104 | -0.0035 | -0.0017 | -0.0002 | --- |  |  |  |
| SG | **0.0319** | **0.019** | **0.0204** | **0.0108** | **0.019** | --- |  |  |
| TK | **0.014** | -0.001 | 0.0015 | -0.0022 | 0.0036 | **0.0159** | --- |  |
| TR | **0.0269** | 0.001 | -0.0017 | -0.0012 | 0.0022 | **0.0256** | 0.004 | --- |

Table S6

| *K* | Mean LnP(*K*) (S.D.) | |
| --- | --- | --- |
|  | Admixture only model | Admixture with location prior |
| 1 | **-87106.65 (0.07)** | -87106.63 (0.06) |
| 2 | -87480.19 (18.65) | **-87085.61 (26.23)** |
| 3 | -88345.01 (85.83) | -87468.00 (50.82) |
| 4 | -91214.68 (219.34) | -88450.07 (233.88) |
| 5 | -95756.50 (507.62) | -89231.73 (499.59) |
| 6 | -98186.58 (1029.24) | -89112.02 (918.24) |
| 7 | -100750.35 (1302.12) | -92778.61 (1732.41) |
